# Balancer-assisted outcrossing to remove unwanted background mutations

**DOI:** 10.17912/micropub.biology.000561

**Published:** 2022-04-28

**Authors:** Katsufumi Dejima, Shohei Mitani

**Affiliations:** 1 Tokyo Women's Medical University

## Abstract

Whole-genome sequencing analysis allows us to identify a large number of natural variants and genetic changes created by mutagenesis. For instance, the Million Mutation Project isolated many point mutant alleles, which are available from the *Caenorhabditis* Genetics Center. Although collections of such mutations are very useful for genetic studies, the strains are often sick because they have multiple other mutations than the mutation of interest. To utilize the strains, it is necessary to outcross with other strains to remove undesired mutations. We previously constructed an inversion balancer toolkit covering a large part of *C. elegans* genome. In contrast to classical translocation balancers that cover parts of two chromosomes, each balancer from the toolkit covers a part of a chromosome. We think this compactness is beneficial for outcrossing mutants containing multiple background mutations. Here, we show that the fluorescence inversion balancer can be practically useful for outcrossing in the case where researchers want to simply evaluate the phenotypes.

**Figure 1. Outcrossing of a Million Mutation Project strain with the fluorescence balancer tmC18[tmIs1200] .**
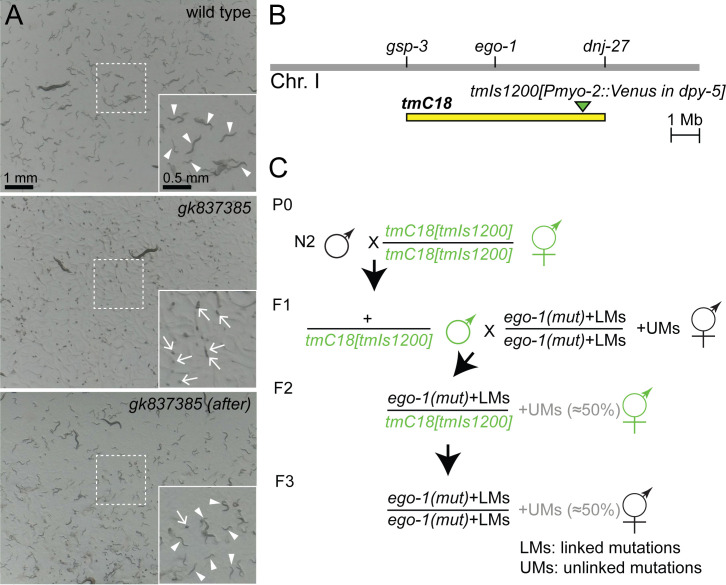
(A) Dissecting microscope view of freshly starved wild type worms (top), *gk837385* worms before outcross (VC40832, middle), and *gk837385* worms after outcross (FX31729, bottom). The panel of the *gk837385* original strain (middle) has many unhatched eggs (arrows). The panels of wild type (top) and the outcrossed strain (bottom) have many small larvae (arrowheads). Insets: enlarged images of the boxed area. (B) Genomic loci covered by *tmC18* . (C) The outcrossing scheme to segregate away unlinked mutations using the fluorescently labeled inversion balancer (shown by green). N2 males are first crossed to the fluorescently labeled inversion balancer strain to generate heterozygous *tmC18[tmIs1200]* /+ males (P0). Males resulting from this cross are then crossed to the *ego-1* mutant hermaphrodites (F1). Venus-positive hermaphrodites resulting from the second cross are then self-fertilized (F2). Following self-fertilization, a non-Venus hermaphrodite is isolated (F3).

## Description

In some situations, researchers may want to evaluate whether mutants of the Million Mutation Project (Thompson *et al.* 2013) show the intended phenotype or not. To utilize the strains, it is required to outcross with other strain to remove background mutations. In the case of deletion mutations, they can be identified by the difference in the size of the PCR bands when genotyping after outcrossing, which is relatively easy. However, the majority of the mutations in this collection are point mutations, and genotyping them by Sanger sequencing and/or restriction fragment length polymorphism (RFLP) analysis are required (Robinson *et al.* 2017). Alternatively, genotyping can be done by using allele-specific PCR (Chen and Schedl 2021) or commercial kits, such as the High Resolution Melting (HRM) analysis (Doyle *et al.* 2021). It should be noted that regardless of the method, one needs to pick and genotype many candidate strains, unless the mutation linked to a visible phenotype. The fluorescent balancer can distinguish heterozygosity of alleles in the covered region: fluorescent marker-negative siblings from the parent bearing mutation over the fluorescent balancer are homozygotes for the mutation of interest because recombination near the locus of interest occurs very rarely (Edgley *et al.* 2006). Theoretically, outcrossing can be achieved by selecting worms without fluorescence for cleaning background mutations not covered by the balancer. Importantly, the balancer-assisted approach does not require genotyping many candidate strains and is most useful in the following situations: (a) the mutation of interest has no visible phenotype, (b) the strain has unlinked deleterious mutations, and (c) there are unlinked mutations that may modify the phenotype of mutation of interest. Indeed, a previous study applied this approach using the *qC1* balancer and provided a good example of the situation (c), where three of *daf-2* mutant strains from the Million Mutation Project also contained *daf-18* mutations that suppress the *daf-2* mutant phenotypes (Bulger *et al.* 2017). We experimentally tested how practically useful outcrossing with a structurally defined inversion balancer that we previously created by CRISPR/Cas9 gene editing (Dejima *et al.* 2018) could be and provide an example where the balancer-assisted approach works on the situation (b).

We focused on the *ego-1* gene, which encodes an RNA-dependent RNA polymerase (Smardon *et al.* 2000). The null mutants for *ego-1* show a sterile phenotype with a germline RNAi defect. When we ordered 23 mutants (see Reagents) from CGC, five strains (VC40084, VC40832, VC40259, VC20439, VC40175) were sick, and 3 of them (VC40084, VC40832, VC40259) were not able to be made frozen stocks on the first receipt. We ordered these three strains from CGC again. Two of the strains were frozen successfully, but one strain (VC40832) was difficult to freeze because it had mostly lethal embryos (Fig. 1A). We performed outcrosses of all strains once using a balancer *tmC18[tmIs1200]* that covers the *ego-1* locus (Fig. 1B and C) (Dejima *et al.* 2018). For 22/23 strains, we obtained healthy worms by outcrossing. For example, for strain VC40832, the outcross improved its embryonic lethality (Fig. 1A, 99.37 ± 0.55 %, n = 20, and 0.18 ± 0.18 %, n = 9, for VC40832 and FX31729, the strain after outcross, respectively). However, for *gk115395* a single outcross did not improve the small reduced brood size (303.8 ± 10.5, n = 4, and 67.6 ± 8.7, n = 4, for N2 and *gk115395* , respectively) suggesting that either additional outcrossing should be performed or that the mutation causing the decreased brood size is covered by the inversion balancer. Alternatively, the reduced brood size could be an *ego-1* phenotype. Importantly, analysis by Sanger sequencing revealed the presence of mutations in all 23 outcrossed strains. Therefore, outcrossing with a fluorescence balancer is practically effective if researchers look for a simple screening method. Although we only tried *tmC18* in this study, one can do the same with other loci and balancers. In addition, while we performed only a single outcross in this study and was able to prepare healthy worms for further experiments, it is important that the process should be repeated to further replace the mutagenized genome with wild type sequences. If the mutants of interest have invisible phenotypes such as behavior abnormality, experiments are greatly enhanced to work with recombination-free balancers as we presented here.

We note that there is a limitation of the balancer-assisted approach: the mutation of interest needs to be covered by one of the existing inversion balancers. The approach is most appropriate for removing unlinked mutations as unwanted mutations that are not covered by the balancer will be removed.

## Methods

-Outcrossing with a balancer

The original *ego-1* mutants (VC strains) were provided by the *Caenorhabditis elegans* Genetics Center, which is supported by the National Institutes of Health National Center for Research Resources. To outcross the strains, males of heterozygotes for *tmC18[tmIs1200]* were crossed with each *ego-1* mutant strain. Venus+ F2 hermaphrodites were singled, then their Venus- F3 progeny was further singled and propagated. To confirm the mutations are present in the outcrossed strains, Sanger sequencing was performed. The primers used for PCR amplification and sanger sequencing are listed in the *ego-1* mutant strain list (see Reagents).

## Reagents

-Balancer strain:

FX30167: *tmC18[tmIs1200(Pmyo-2::Venus)] I*

*-ego-1* mutant strains:

**Table:** 

Original strain	allele	position	Outcrossed strain	Primers (PCR, Sanger seq.)
VC40259	*gk540555*	7650738	FX31713	F26A3#F37R40, R40
VC40886	*gk864727*	7650888	FX31714	F26A3#F37R41, R41
VC20206	*gk115390*	7651213	FX31715	F26A3#F38R42, F38
VC20319	*gk317493*	7651478	FX31716	F26A3#F39R43, F39
VC30058	*gk115391*	7651882	FX31717	F26A3#F40R21, F40
VC40244	*gk532049*	7652169	FX31718	F26A3#F41R21, F41
VC40660	*gk749674*	7652199	FX31719	F26A3#F41R21, F41
VC41006	*gk925207*	7652313	FX31720	F26A3#F41R21, F41
VC20618	*gk357146*	7652424	FX31721	F26A3#F41R21, F41
VC40613	*gk721963*	7653391	FX31722	F26A3#F19R21, F44
VC40920	*gk882383*	7653502	FX31723	F26A3#F19R1, F44
VC40140	*gk481348*	7653535	FX31724	F26A3#F19R1, F44
VC20439	*gk115393*	7653805	FX31725	F26A3#F19R1, F45
VC40951	*gk896494*	7653809	FX31726	F26A3#F19R1, F45
VC30158	*gk426642*	7654398	FX31727	F26A3#F19R1, R1
VC20474	*gk115394*	7654992	FX31728	F26A3#F18R44, R44
VC40832	*gk837385*	7655076	FX31729	F26A3#F18R45, F18
VC40084	*gk115395*	7655149	FX31730	F26A3#F18R46, R46
VC20545	*gk115397*	7655230	FX31731	F26A3#F18R47, R47
VC40175	*gk498425*	7655401	FX31732	F26A3#F18R47, R47
VC40116	*gk470185*	7655705	FX31733	F26A3#F18R48, R48
VC40611	*gk720210*	7656073	FX31734	F26A3#F42R12, F42
VC40050	*gk115401*	7656215	FX31735	F26A3#F43R12, F43

-Oligonucleotides:

**Table:** 

Primer name	Sequence 5’ > 3’
F26A3#F18	AAGCTCCACGAACTGTCATC
F26A3#F19	AGGTGGAATCATTTCGCCAG
F26A3#F37	GTTCCGACATGACGAGGAGT
F26A3#F38	CTACCAGCTTGCGATGAACC
F26A3#F39	GTAGTAGGTTTTGAGTGCGG
F26A3#F40	CGTGCCCATTCGTCTAACAT
F26A3#F41	TTACTATCACGTCCGTAACG
F26A3#F42	CGACGTCTAACGTCATCCCA
F26A3#F43	ACCTGCTGGTCGCAACTTCA
F26A3#F44	CCAAGCATTGACCGTCCGAG
F26A3#F45	GAAAGCATTTGTCTGTCCAG
F26A3#R1	GGAATATGGCACCGATTCCT
F26A3#R12	GTCACGTTCTGCTTCCATCT
F26A3#R21	TCGCCAGTTCCAGTGGCATT
F26A3#R40	GGTTCATCGCAAGCTGGTAG
F26A3#R41	CCGCACTCAAAACCTACTAC
F26A3#R42	ATGTTAGACGAATGGGCACG
F26A3#R43	TCGCATATGACGTCATCGCA
F26A3#R44	AGCGCGAATCAGTATCCAAC
F26A3#R45	GGAATCGGTGAGCACACCCA
F26A3#R46	TAGAAGAGCCTACCGAACGG
F26A3#R47	CGTTAGACGTCGGAAGTCGG
F26A3#R48	ATTGCGGCGATCCAGACATA
