# Corrigendum: Balancer-assisted outcrossing to remove unwanted background mutations

**DOI:** 10.17912/micropub.biology.000572

**Published:** 2022-05-10

## Description

For Dejima, K; Mitani, S (2022). Balancer-assisted outcrossing to remove unwanted background mutations. microPublication Biology. 10.17912/micropub.biology.000561.

The authors correct the following:

In the reference section, the PubMedID for Dejima et al., is PubMed ID: 29298424 instead of PubMed ID: 34140407

Dejima K, Hori S, Iwata S, Suehiro Y, Yoshina S, Motohashi T, Mitani S. 2018. An Aneuploidy-Free and Structurally Defined Balancer Chromosome Toolkit for Caenorhabditis elegans. Cell Rep 22: 232-241. PubMed ID: 29298424.

